# Enhanced bioproduction of phenolic compounds from *Populus nigra* L. using temporary immersion bioreactors and metabolomics

**DOI:** 10.3389/fmolb.2025.1704160

**Published:** 2025-11-24

**Authors:** Malorie Laffon, Florent Magot, Caroline Birer Williams, Franck Michoux, Clément Lemoine, Arnaud Lanoue, Nathalie Giglioli-Guivarc’h

**Affiliations:** 1 UR 2106 Biomolécules et Biotechnologies Végétales, Faculté de Pharmacie, Université de Tours, Tours, France; 2 Evonik Advanced Botanicals, Parçay-Meslay, France

**Keywords:** black poplar, specialized metabolites, plant biotechnology, temporary immersion system, elicitation, metabolomics, bioproduction

## Abstract

Black poplar is a woody species rich in bioactive phenolic compounds with promising pharmaceutical and cosmetic applications. In wild populations, genetic diversity and environmental variability affect phenolic content and bioactivities. Plant tissue culture, conducted under controlled conditions, offers a suitable alternative for industrial bioproduction. The aim of this study was the development of *in vitro* shoot cultures of black poplar in Magenta™ and RITA® systems for efficient and constant phenolic productivity. Following the initiation of *in vitro* lines, significant growth enhancement was achieved through selection of fast-growing lines. UPLC-QTOF-MS-based untargeted metabolomic analyses were carried out, and allowed the identification of flavan-3-ols, proanthocyanidins, flavonols and salicinoids. As a result, 32 compounds were described for the first time in *P. nigra*, including 15 metabolites previously identified in other *Populus* species and 17 additional compounds not yet identified in the *Populus* genus. The productivity of the major phenolic compounds was substantially higher in the RITA® system, showing a 2.6-fold increase compared to Magenta™. Targeted metabolomics followed by Principal Component Analysis were performed to study the metabolic changes during 8 weeks of culture in RITA® system, revealing that most metabolites accumulated during the three first weeks of growth. Optimal shoot growth and phenolic content were achieved in RITA® system after 3 weeks of culture. Finally, the effects of both SA and MeJa treatments were characterized by Orthogonal Partial Least Squares Discriminant Analysis (OPLS-DA) which identified metabolites with Variable Importance in Projection (VIP >1), notably flavan-3-ols and proanthocyanidins as biomarkers common to both elicitation treatments. In conclusion, *in vitro* shoot culture in RITA® system and metabolomics studies allowed to design a specific process for efficient bioproduction of black poplar bioactive phenolics.

## Introduction

Black poplar (*Populus nigra*), is a tree from the Salicaceae family, widely distributed in Europe, Asia and Northern Africa. The genus *Populus* consists of 29 species, divided into six sections (Eckenwalder et al., 1996). Among all these species, black poplar revealed promising applications compared to other *Populus* species. Traditionally, *P. nigra* buds were used to prepare ointments for the treatment of inflammations and hemorrhoids (Gerarde, 1597).

Black poplar extracts exhibit biological activities sought after by pharmaceutical and cosmetic industries. Among these activities, *in vitro* antioxidant properties of bud extracts have been reported (Dudonné et al., 2011), as well as anti-inflammatory (Pobłocka-Olech et al., 2019) and antimicrobial (Kis et al., 2022) properties. Furthermore, in the literature, *P. nigra* extracts exhibited higher biological activities compared to other species from the *Populus* genus. For instance, regarding antioxidant activities, *P. nigra* extracts revealed higher activity than *Populus balsamifera* (Stanciauskaite et al., 2021). Also, *P. nigra* exhibits stronger antibacterial activities toward several Gram-positive bacteria than *P. tremula* (Benedec et al., 2014). Similar results have also been reported when compared to *Populus alba*, particularly for antibiofilm activity (Nassima et al., 2019).

**FIGURE 1:**
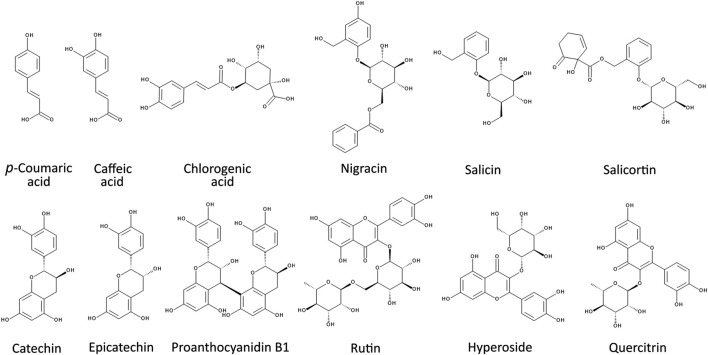
Chemical structures of selected specialized metabolites identified in *P. nigra* extracts.

*P. nigra* exhibits a rich diversity of specialized metabolites that likely contribute to its biological activities. These metabolites include a wide range of phenolic compounds such as salicinoids, anthocyanins, flavan-3-ols, condensed tannins, phenolic acids, as well as various terpenoids including hemiterpenes, sesquiterpenes and monoterpenes (Chen et al., 2009; Devappa et al., 2015; Palo, 1984) (Figure 1). The distribution of these metabolites is organ-specific. For instance, the bark is rich in salicinoids (Palo, 1984), while flowers contain high levels of anthocyanins (Alcalde-Eon et al., 2016) and the stems accumulate flavan-3-ols and condensed tannins (Ullah et al., 2019a). In the same way, volatile compounds including hemiterpenes, sesquiterpenes and monoterpenes are released specifically by the buds (Jerkovic and Mastelic, 2003). Among the different plant parts, the leaves and buds have received more attention in phytochemical studies as they are especially rich in phenolic compounds such as flavonoids, phenolic acids and salicinoids (Kuś et al., 2018; Pobłocka-Olech et al., 2021).

The production of specialized metabolites in *Populus* species can vary significantly depending on the type of culture and environmental conditions. Indeed, such metabolites play essential roles in mediating plant-environment interactions serving as part of the plant’s adaptive defense mechanisms against various biotic and abiotic stresses. For example, concentrations of terpenes and salicin increase in response to herbivore attacks, salicinoids accumulate under drought conditions, proanthocyanidins increase following fungal infections, and flavonoid levels increase after exposure to UV-B radiations (Fabisch et al., 2019; Hale et al., 2005; Miranda et al., 2007; Warren et al., 2023).

The black poplar extracts used as active ingredients in phytotherapy or cosmetics are primarily derived from field-grown plants, which are subjected to various environmental constraints. Indeed, these biotic and abiotic factors may significantly influence the phenolic metabolism, leading to annual variations of bioactive compounds in the extracts, thereby hindering standardization and large-scale production. To overcome these limitations, plant tissue culture techniques have been developed since the beginning of the 20th century (Haberlandt, 1902). *In vitro* plant technologies, conducted under controlled sterile conditions, enable the homogeneous and steady production of biomass and specialized metabolites. For example, several specialized metabolites of high pharmaceutical relevance are produced *in vitro*, such as paclitaxel and podophyllotoxin, two molecules used for cancer treatment and obtained from *Taxus* and *Podophyllum* species respectively (Espinosa-Leal et al., 2018). Line selection and optimization of dedifferentiated cell cultures, such as cell suspension or callus cultures, have enabled the accumulation of specialized metabolites sometimes higher than in whole plants (Costa et al., 2013; Nagella and Murthy, 2010; Reis et al., 2018). However, sometimes, cell differentiation was shown to play a crucial role in the biosynthesis of specialized metabolites in specific organs (Besseau et al., 2013; Guo et al., 2022; Liu et al., 2020; Xu et al., 2018).

To enable the scale-up of organ and tissue cultures, Temporary Immersion Systems (TIS) have been developed, offering high biomass and metabolite production compared to conventional solid and liquid culture methods (Mirzabe et al., 2022). For example, temporary immersion cultures of *Ananas comosus* produced higher biomass compared to liquid cultures, by enhanced oxygen uptake and improved assimilation of sugar, nitrate and ammonium (Escalona et al., 2003). Similarly, TIS improved the development of *Bletilla striata*, increasing stem diameter, plant height and leaf width, compared to solid cultures (Zhang et al., 2018). In *Vitis flexuosa,* TIS also allowed higher plant height and root length, as well as higher contents in total flavonoids and total phenolics (Park et al., 2015). To further enhance the specialized metabolite production, elicitation techniques are commonly used to induce a defense responses (Selwal et al., 2024). For instance, successful elicitation experiments in TIS led to increased levels of lignans in *Schisandra rubriflora* and *Schisandra chinensis* (Szopa et al., 2022; 2018), phenolic compounds in *Centella asiatica* (Skrzypczak-Pietraszek et al., 2019) and alkaloids in *Dendrobium nobile* (Cao et al., 2024; Zhang B et al., 2022).

Despite the reported biological activities of black poplar, *in vitro* cultures have not yet been established for the efficient production of specialized metabolites. To date, phytochemical studies on black poplar specialized metabolites have focused exclusively on wild and greenhouse-grown cultures of black poplar, with no available data on metabolite production from *in vitro* cultures. Previous *in vitro* cell and organ cultures of *P. nigra* were initially developed to study osmotic stress responses or phytoremediation potential (Busont et al., 2023; Iori et al., 2012a; 2012b).

This study aims to develop, for the first time, the bioproduction of black poplar-derived bioactive compounds for cosmetic applications. To reach this objective, *in vitro* shoot cultures were initiated from black poplar buds and fast-growing lines were selected for further optimization. Subsequently, untargeted High-Resolution Mass Spectrometry (HRMS) analyses were conducted to characterize the metabolomic profiles of *in vitro* extracts. Growth and metabolite production of *P. nigra* cultures grown in TIS were assessed for 8 weeks to determine the optimal period for elicitation. Then, cultures were treated with methyl jasmonate (MeJa) or salicylic acid (SA) to induce the production of specialized metabolites of interest. Finally, targeted metabolomics and multivariate analysis were performed to identify the metabolites and quantify the effect of elicitation.

## Materials and methods

### Chemicals and reagents

Murashige and Skoog (MS) medium, 6-benzylaminopurine (BAP), naphtaleneacetic acid (NAA), Gelrite™, sucrose and methyl jasmonate (MeJa) were supplied by Duchefa Biochemie BV (Haarlem, Netherlands). Mannitol and ethanol for *in vitro* culture purposes were purchased from VWR (Radnor, PA, USA). Ethanol for phenolic extraction, as well as methanol and acetonitrile were of analytical grade and supplied by Fisher Scientific (Schwerte, Germany). Salicylic acid (SA), mercury chloride, formic acid and the standards catechin, epicatechin, salicin and nigracin were provided by Merck (Darmstadt, Germany). Sodium hydroxide was purchased from Honeywell (Charlotte, NC, USA). The standards procyanidin B1 and B3, caffeic acid, isoquercitrin, quercitrin, rutin and hyperoside were supplied by Extrasynthèse (Genay, France), populoside by Biosynth (Staad, Switzerland), salicortin by PhytoLab (Vestenbergsgreuth, Germany), 3-O-caffeoylquinic acid (CQA), 4-CQA and 5-CQA by Toronto Research Chemicals (Toronto, Canada). *p*-Coumaric acid and quercetin were purchased from Sigma-Aldrich (St Louis, MI, USA).

### Plant materials and initiation of cultures

*Populus nigra L.* plants were bought from Naudet Pépinières (Yonne, France) and authenticated by barcoding identification by DNA Gensee (Le Bourget du Lac, France). Nodal segments from the saplings were cut for the decontamination of axillary buds. The sterilization protocol was briefly optimized, by testing the following conditions: washing with sterile water and/or ethanol 70%, followed by decontamination with NaClO at 1% or 1.2%, or HgCl_2_ at 0.1% or 0.2%. A successful decontamination percentage of 100% was obtained by washing with ethanol 70% for 30 s, followed by 15 min in NaClO 1%, and rinsing several times in sterile water. The nodes were placed on solid culture medium composed of Murashige and Skoog basal salts and vitamins, supplemented with 30 g/L sucrose and 2.5 g/L Gelrite™, adjusted to a pH of 5.8 (Murashige and Skoog, 1962). Cultures were maintained at 25 C with a 16-h photoperiod with LED lighting at an intensity of 70 μmol/s/m^2^. After 2 months, explants were subcultured on MS medium supplemented with 30 g/L sucrose, 2.5 g/L Gelrite™, 0.1 mg/L BAP and 0.02 mg/L naphtaleneacetic acid (NAA), adjusted to a pH of 5.8 (Whitehead and Giles, 1977). Cultures were grown in Magenta™ vessels, containing five explants per box, maintained under the same conditions as described previously, with subculturing onto fresh medium every 2 weeks.

### Line selection

After initiation of cultures, three lines were obtained from the original saplings called PN1, PN2 and PN5. Their multiplication rates were compared to identify the most vigorous and fast-growing line. Multiplication rate is defined as the number of new explants obtained from one explant when subculturing every 2 weeks.

Liquid cultures of lines PN1, PN2 and PN5 were established in Automated Temporary Immersion Recipients (RITA®) (Vitropic, Saint-Mathieu de Tréviers, France). For growth comparison, five explants of each line were inoculated in RITA® containing 200 mL of liquid culture medium containing MS basal medium supplemented with 30 g/L sucrose, 0.1 mg/L BAP and 0.02 mg/L NAA, adjusted to a pH of 5.8. The explants, 2 weeks old and intact, were transferred into the RITA® bioreactors. Cultures were maintained at 25 C with a 16-h photoperiod under LED lighting at an intensity of 70 μmol/s/m^2^. Immersion cycles were set to 5 min every 4 h. Each line was cultivated in triplicate. After 4 weeks of growth, biomass was harvested, and the Growth Index (GI) based on dry weight (DW) was calculated as follows:

$$GI=\frac{Harvested\;biomass\;\left(g\right)}{Inoculated\;biomass\;\left(g\right)}$$

### Study of growth kinetics in RITA® bioreactors

For analysis of growth kinetics, 2-week-old explants of line PN5 were cut, and 3 g of biomass were inoculated in RITA® bioreactor. Harvests were performed weekly over a period of 8 weeks, with three biological replicates per time point. Fresh biomass was weighed immediately after harvest. For dry weight (DW) estimation, the biomass was first blotted on paper to remove the excess surface moisture, then frozen, freeze-dried with Labconco® Freezone freeze dryer (Labconco, Kansas City, MO) and subsequently weighed.

### Elicitation experiments

For elicitation experiments, RITA® bioreactors were inoculated with 5 g (FW) of line PN5 explants and maintained as described previously. After 3 weeks of growth, cultures were elicited with methyl jasmonate (MeJA) or salicylic acid (SA). Stock solutions of MeJA and SA were prepared at 100 mM in absolute ethanol and distilled water, respectively, and added to the culture medium to reach a final concentration of 100 µM. Treatments were performed in triplicate, with samples harvested after 1, 2, 3 and 4 days. Control cultures were harvested in four replicates after 1 and 4 days. Fresh and dried biomass were obtained as mentioned previously.

### Specialized metabolite extraction

The dried biomass was ground to a fine powder using a grinding mill (A10 basic, IKA®) and stored at 4 C until extraction. Twenty milligrams of powder were extracted in 1 mL of 70:30 (v/v) ethanol/water using a sonication bath, at room temperature, for 30 min. The extracts were centrifuged for 10 min at 16,900 g. For untargeted metabolomic analyses by UPLC-ToF-MS, the supernatant was diluted 1:50, while a 1:5 dilution was used for targeted phenolic quantification by UPLC-MS, using a solution constituted of 5:95 (v/v) acetonitrile/water. Extracts were stored at 4 C until analysis. All extractions were performed in triplicate.

### UPLC-MS analyses

Untargeted metabolomic analyses were carried out on an ACQUITY™ Ultra Performance Liquid Chromatography (UPLC) system coupled to a high-resolution QToF mass spectrometer (SYNAPT G2-Si, Waters Corporation, Milford, USA). Analyte separation was performed using a Waters Acquity HSST3 C_18_ column (150 × 2.1 mm, 1.8 µm) with a flow rate of 0.4 mL/min and an injection volume of 1 µL. The column was heated at 55 C and the autosampler temperature was set at 8 C. The mobile phase consisted of solvent A (0.1% formic acid in water) and solvent B (0.1% formic acid in acetonitrile). Chromatographic separation was achieved using an 18-min elution gradient as follows: 0 min, 97:3 (A:B); 18 min, 60:40; 18.1 min, 5:95; 21.1, min 5:95; 21.2 min, 97:3; 26 min, 97:3. Mass spectrometry detection was performed in negative mode with Fast-DDA acquisition, consisting of a full MS survey scan from 50 to 1200 m/z (scan time = 0.1 msec) followed by MS/MS scans for the three most intense ions (m/z 100–1200; scan time = 0.1 msec). A collision energy ramp was set from 10 to 40 eV for low masses and 40–90 eV for high masses. Files were transformed into mzML format with MSConvert software, part of the ProteoWizard package (Chambers et al., 2012). Data were treated with the softwares MZmine version 3.9.0 (Schmid et al., 2023) and SIRIUS version 5.8.6 (Dührkop et al., 2019) for features detection, alignment and molecular formula generation and putative annotation, with comparison of MS/MS spectra comprised in GNPS repositories (Wang et al., 2016), in the existing literature and/or with MS/MS scans of authentic standards when available. As defined by the Metabolomics Standard Initiative (Blaženović et al., 2018), different degrees of confidence in compound identification were distinguished: Level 1 (confident 2D structure) involves matching two orthogonal pieces of information such as retention time (RT), accurate mass and MS/MS data (accurate mass and fragmentation pattern) with a standard. Level 2 (probable structure) is also based on two orthogonal pieces of analytical evidence but by comparison with bibliographic data or databases through diagnostic evidence. Level 3, denoting that one or several candidates are possible, requires at least one piece of information supporting the proposed candidate (*e.g.*, by comparison of their MS data only).

Targeted metabolomics for phenolic quantification was carried out using an ACQUITY™ H-Class UPLC system coupled to a photo diode array detector (PDA) and a Xevo TQD mass spectrometer (Waters Corporation, Milford, USA) equipped with an electrospray ionization (ESI) source controlled by Masslynx 4.2 software (Waters Corporation, Milford, USA). MS detection was performed in both positive and negative modes, with *m*/*z* ratios from 50 to 2000. The capillary voltage was 2000 V and sample cone voltages were 30 and 50 V. The desolvation gas flow rate was 1000 L h^−1^.

Standards of hyperoside, catechin, epicatechin, proanthocyanidins B1 and B3, 5-caffeoylquinic acid, 3-caffeoylquinic acid, salicin, caffeic acid, *p*-coumaric acid, rutin, isoquercitrin, salicortin, quercitrin, nigracin and populoside were injected at 0.5, 1, 2, 5, 8 and 10 ppm for absolute quantification. Concentration of the unknown proanthocyanidin dimer was expressed in equivalents of proanthocyanidin B1.

### Statistical analysis

All data were treated using Minitab software (Minitab 1nc., USA). Results are expressed as mean ± standard deviation (SD). Statistical differences were revealed with a Kruskal–Wallis test followed by multiple comparisons with a Bonferroni correction. Statistically different groups (*p < 0.05*) are represented by different letters. Multivariate Statistical Analysis (MVA) were performed using the software SIMCA® 18 (Sartorius AG, Göttingen, Germany). All variables were mean-centered, and unit-variance (UV) scaled prior to MVA. Orthogonal Partial Least Squares Discriminant Analysis (OPLS-DA) were performed according to elicitation treatments to identify the Variable Important in Projection (VIP >1).

## Results

### Establishment of *P. nigra* axenic cultures

Black poplar axillary buds were successfully decontaminated in ethanol 70% for 30 s and sodium hypochlorite 1% for 15 min. Subculture of decontaminated explants on multiplication medium described above led to the acquisition of three poplar lines called PN1, PN2 and PN5, each derived from a different bud. Line selection is necessary to ensure optimal multiplication rate and growth for large-scale *in vitro* plant biomass production.

In Magenta™ vessels, line PN5 showed the highest multiplication rate after 2 weeks (2.6 ± 0.5), compared to PN1 (1.5 ± 0.1) and PN2 (1.8 ± 0.4) (Supplementary Figure S1A). After transferring the explants into RITA® systems, PN5 also had the highest growth index after 4 weeks (2.6-fold), compared to PN1 (1.9-fold) and PN2 (1.4-fold) (Supplementary Figure S1B). Based on its performance in both culture systems, line PN5 was selected for further experiments.

Its growth kinetics in RITA® systems was then monitored over 8 weeks to determine the optimal time for elicitation, when explants are still actively growing.

**FIGURE 2:**
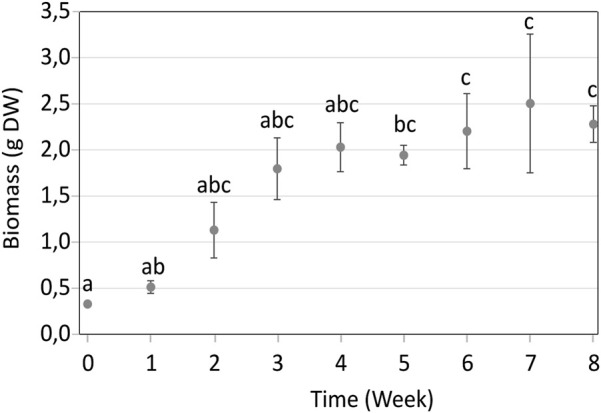
Growth kinetics of *in vitro Populus nigra* shoots cultivated in RITA® systems over 8 weeks. Different letters indicate significant differences (p < 0.05).

**FIGURE 3:**
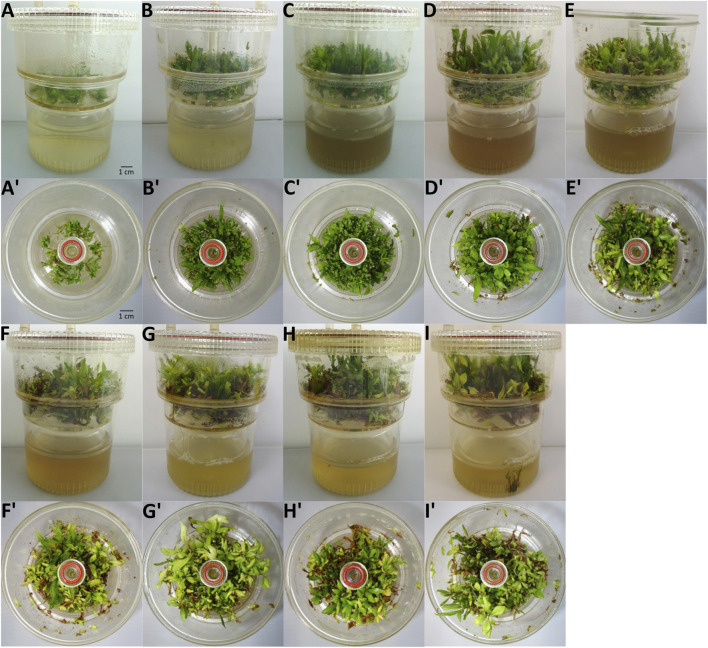
Morphological appearance of shoot development in RITA® systems over the 8 weeks of growth. **(A–I)** Side view from week 0 to week 8. **(A′-I′)** Top view from week 0 to week 8.

The growth of *P. nigra* shoots in RITA® systems increased steadily until the fourth week of culture, then slowed down between weeks four and 8 (Figure 2). Moreover, after week 4, the old-explant leaves began turning yellow and brown, showing early signs of wilting, while young shoots were green (Figure 3). This browning spread on all explants after week 8. Therefore, week three was selected as the optimal time for elicitation, as it corresponded to the highest growth index with explants still appearing healthy and without browning. At this stage, biomass reached 1.80 g of DW, corresponding to a growth index of 5.5.

### Untargeted metabolic profiling of *P. nigra in vitro* shoots

**FIGURE 4:**
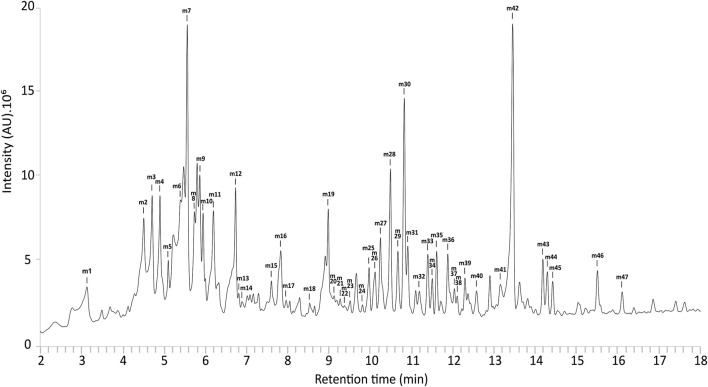
UV chromatogram of extracts from *Populus nigra* shoots grown in Magenta™ systems.

UPLC-MS metabolic profiling of line PN5 grown in Magenta™ vessels revealed a UV chromatogram with a rich diversity in specialized metabolites (Figure 4). UPLC-HRMS based acquisitions served for feature detection within samples, molecular formula generation and MS/MS comparison with MS/MS data of pure compounds or/and MS/MS data from the available literature to putatively identify compounds (Table 1). Out of 47 detected features, 37 were identified or putatively identified, including eight phenolic acids, 2 flavan-3-ols, three condensed tannins, 17 salicinoids and seven flavonols. Black poplar shoots grown in RITA® bioreactors showed an identical profile to shoots grown in Magenta™ vessels (data not shown).

**TABLE 1:** UHPLC-ESI-HRMS data of extracts from *Populus nigra* shoots grown in Magenta™ system. Numbers in the column “ID” refer to peak numbers in Figure 4. *According to Blaženović et al., 2018.

Compound class	ID	RT (min)	λ_max_ (nm)	Compound assignment	m/z measured	MS/MS fragments ES^-^	Molecular formula	Error (ppm)	Level of identification*	References
Phenolic acids	m1	3.14	212	Dihydroxybenzoic acid hexoside	315.0709	153.0177 [M-H-C_6_H_10_O_5_]^−^	C_13_H_16_O_9_	−3.9	3	Li et al. (2014)
	m2	4.51	213	5-O-Caffeoylquinic acid	353.0877	191.0529 [M-H-C_9_H_6_O_3_]^−^	C_16_H_18_O_9_	−0.2	1	Standard
	m4	4.9	215/330	Caffeic acid hexoside	341.0874	179.0348 [M-H-C_6_H_10_O_5_]^−^, 161.0223 [M-H-C_6_H_10_O_5_-H_2_O]^−^	C_15_H_18_O_9_	0.42	3	Saleme et al. (2017)
	m8	5.74	205/283	3-O-Caffeoylquinic acid	353.0868	191.0550 [M-H-C_9_H_6_O_3_]^−^	C_16_H_18_O_9_	−1.29	1	Standard
	m10	5.95	218/315	Coumaric acid glucoside	325.0916	145.0282 [M-H-C_6_H_10_O_5_-H_2_O]^−^	C_15_H_18_O_8_	−2.28	2	Morse et al., 2007; Wang et al., 2018
	m11	6.2	219/292	Caffeic acid	179.0356		C_9_H_8_O_4_	6.34	1	Standard
	m12	6.73	218/329	Ferulic acid hexoside	355.1029	193.0501 [M-H-C_6_H_10_O_5_]^−^, 175.0403 [M-H-C_6_H_10_O_5_-H_2_O]^−^	C_16_H_20_O_9_	−0.02	3	Spínola et al. (2015)
	m16	7.83	218/310	p-Coumaric acid	163.0403		C_9_H_8_O_3_	4.61	1	Standard
Salicinoids	m3	4.71	212	Salicin	285.0972	331.1028 [M-H+CH_2_O_2_]^−^, 123.0451 [M-H-C_6_H_10_O_5_]^−^	C_13_H_18_O_7_	−0.80	1	Standard
	m22	9.37	218	Salicortin	423.1292	469.1341 [M-H+CH_2_O_2_]^−^, 155.0384 [M-H-C_13_H_16_O_6_]^−^, 123.0442 [M-H-C_13_H_16_O_8_]^−^	C_20_H_24_O_10_	0.18	1	Standard
	m26	10.11	218/327	Populoside isomer	447.1292	323.0746 [M-H-C_7_H_6_O_2_]^−^, 179.0333 [M-H-C_13_H_16_O_6_]^−^, 161.0205 [M-H-C_13_H_16_O_6_-H_2_O]^−^	C_22_H_24_O_10_	0.12	3	Okińczyc et al. (2024); Zhang et al. (2006)
	m27	10.24	218/327	Grandidentoside	439.1596	179.0349 [M-H-C_12_H_20_O_6_]^−^, 161.0224 [M-H-C_12_H_20_O_6_-H_2_O]^−^	C_21_H_28_O_10_	−1.87	2	Abreu et al. (2020); Erickson et al. (1970)
	m30	10.82	225/282	Nigracin	405.1180	451.1236 [M-H+CH_2_O_2_]^−^	C_20_H_22_O_9_	−1.38	2	Standard
	m31	10.91	218/325	Populoside isomer	447.1292		C_22_H_24_O_10_	0.12	3	Okińczyc et al. (2024); Zhang et al. (2006)
	m33	11.39	218/314	Trichocarposide	431.1331		C_22_H_24_O_9_	−3.8	2	Pearl and Darling (1971); Sobeh et al. (2019)
	m35	11.61	219/314	Isograndidentatin A	423.1669		C_21_H_28_O_9_	2	2	Si et al. (2011); Tawfeek et al. (2019)
	m36	11.88	219/314	Grandidentatin A	423.1656	163.0403 [M-H-C_12_H_20_O_6_]^−^, 145.0285 [M-H-C_12_H_20_O_6_-H_2_O]^−^	C_21_H_28_O_9_	0.22	2	Si et al. (2011); Tawfeek et al. (2019)
	m37	12.03	219/324	Populoside	447.1274		C_22_H_24_O_10_	−3.85	1	Standard
	m39	12.29	219/314	Populoside B	431.1334		C_22_H_24_O_9_	−1.93	2	Zhang et al. (2006)
	m42	13.45	225/284	Homaloside D	543.1494	589.1545 [M-H+CH_2_O_2_]^−^, 405.1171 [M-H-C_7_H_6_O_3_]^−^, 387.1093 [405-H_2_O]^−^	C_27_H_28_O_12_	−1.57	2	Boeckler et al. (2013); Ekabo and Farnsworth (1993)
	m45	14.41	220	Populin	389.1228		C_20_H_22_O_8_	−2.17	2	Pobłocka-Olech et al. (2021); Van Hoof et al. (1989)
Proanthocyanidins	m5	5.11	205	Procyanidin B1	577.1340	289.0703 [M-H-C_15_H_12_O_6_]^−^	C_30_H_26_O_12_	−1.04	1	Standard
	m6	5.4	205	Procyanidin B3	577.1304		C_30_H_26_O_12_	−7.31	1	Standard
	m15	7.6	212	Procyanidin B	577.1319		C_30_H_26_O_12_	−4.68	3	Piccinelli et al. (2016)
Flavan-3-ols	m7	5.56	201	(+)-Catechin	289.0719		C_15_H_14_O_6_	2.38	1	Standard
	m13	6.81	209	(−)-Epicatechin	289.0716		C_15_H_14_O_6_	1.25	1	Standard
Flavonols	m19	8.98	212/351	Rutin	609.1461	301.0358 [M-H-C_12_H_20_O_9_]^−^	C_27_H_30_O_16_	0.89	1	Standard
	m20	9.12	218	Hyperoside	463.0845		C_21_H_20_O_12_	−6.80	1	Standard
	m21	9.27	218	Isoquercitrin	463.0883		C_21_H_20_O_12_	1.34	1	Standard
	m24	9.81	218	Isorhamnetin hexoside	477.1050		C_22_H_22_O_12_	3.50	3	Danise et al., 2021; Pobłocka-Olech et al., 2021
	m25	9.97	219/321	Kaempferol rutinoside	593.1486		C_27_H_30_O_15_	−3.48	2	Danise et al. (2021)
	m28	10.49	218/330	Quercitrin	447.1280	301.0356 [M-H-C_6_H_10_O_5_]^−^			1	Standard
	m29	10.67	219	Isorhamnetin hexoside	477.1030		C_22_H_22_O_12_	−0.68	3	Danise et al. (2021); Pobłocka-Olech et al. (2021)
Flavanones	m43	14.18	220/283	Pinostrobin pentoside hexoside	563.1772	609.1820 [M-H+CH_2_O_2_]^−^, 269.0812 [M-H-C_5_H_8_O_4_-C_6_H_10_O_5_]^−^	C_27_H_32_O_13_	1.26	3	Ristivojević et al. (2015); Rubiolo et al. (2013)
	m44	14.28	220/284	Pinostrobin pentoside hexoside	563.1769	609.1807 [M-H+CH_2_O_2_]^−^, 269.0818 [M-H-C_5_H_8_O_4_-C_6_H_10_O_5_]^−^	C_27_H_32_O_13_	0.72	3	Ristivojević et al. (2015); Rubiolo et al. (2013)
	m46	15.5	220/283	Pinostrobin hexoside	431.1334	269.0817 [M-H-C_6_H_10_O_5_]^−^	C_22_H_24_O_9_	−3.1	3	Ristivojević et al. (2015); Rubiolo et al. (2013)
	m47	16.1	220	Pinostrobin hexoside	431.1371	269.0811 [M-H-C_6_H_10_O_5_]^−^	C_22_H_24_O_9_	−6.95	3	Ristivojević et al. (2015); Rubiolo et al. (2013)

Sixteen compounds were identified with confidence by comparison with pure authentic standards (level 1 of identification) corresponding to 5-caffeoylquinic acid (m2), salicin (m3), procyanidin B1 (m5), procyanidin B3 (m6), catechin (m7), 3-caffeoylquinic acid (m8), caffeic acid (m11), epicatechin (m13), *p*-coumaric acid (m16), rutin (m19), hyperoside (m20), isoquercitrin (m21), salicortin (m22), quercitrin (m28), nigracin (m30) and populoside (m37).

Other analytes were putatively identified with a confidence level 2 or three of identification. Compound m1 produced the following ions in negative ionization; [M-H]^-^ at *m*/*z* 315.0709 and [M-H-hexoside]^-^ at *m*/*z* 153.0177. Accurate mass measured with high resolution analysis closely matches the molecular formula C_13_H_16_O_9_ that corresponds to dihydroxybenzoic acid hexoside (Li et al., 2014). Compound m4 produced the following ions in negative ionization; [M-H]^-^ at *m*/*z* 341.0874, [M-H-hexoside]^-^ at 179.0348 and [M-H-hexoside-H_2_O]^-^ at *m*/*z* 161.0223. These MS features led to the identification of a caffeic acid hexoside, as previously described in *P. tremula x P. alba* (Saleme et al., 2017). Metabolite m10 produced a [M-H]^-^ ion at *m*/*z* 325.0916 in ES^−^ and a [M-H-glucose-H_2_O]^-^ ion at *m*/*z* 145.0282, corresponding to coumaric acid glucoside as previously described in *Vaccinium macrocarpon* (Wang et al., 2018). Metabolite m12 produced a [M-H]^-^ ion at *m*/*z* 355.1029, a [M-H-glucose]^-^ at *m*/*z* 193.0501 and a [M-H-glucose-H_2_O]^-^ ion at *m*/*z* 175.0403 in ES^−^. These MS features correspond to ferulic acid glucoside (Spínola et al., 2015). Compounds m26 and m31 produced [M-H]^-^ at *m*/*z* 447.1292 in ES^−^, as well as [M-H-C_7_H_6_O_2_]^-^ at *m*/*z* 323.0746, [M-H-C_13_H_16_O_6_]^-^ at *m*/*z* 179.0333 and [M-H-C_13_H_16_O_6_-H_2_O]^-^ at *m*/*z* 161.0205 for m26, corresponding to populoside isomers as already described in the *Populus* genus (Okińczyc et al., 2024; Zhang et al., 2006). Metabolite m27 presented [M-H]^-^ at *m*/*z* 439.1596, [M-H-C_12_H_20_O_6_]^-^ at *m*/*z* 179.0349 and [M-H-C_12_H_20_O_6_-H_2_O]^-^ at *m*/*z* 161.0224; corresponding to grandidentoside as previously described in *Populus tremula* (Abreu et al., 2020). Compound m33 presented the ion [M-H]^-^ at *m*/*z* 431.1331. The accurate mass measurement and retention time were compared with the literature and led to the annotation of the compound as the salicinoid trichocarposide, previously described in *Populus* and *Salix* species (Pearl and Darling, 1971; Sobeh et al., 2019). Compounds m35 and m36 showed similar molecular ion high-resolution masses with ions [M-H]^-^ respectively at *m*/*z* 423.1669 and 423.1656. Compound m36 also showed fragments at 163.0403 corresponding to [M-H-C_12_H_20_O_6_]^-^, and 145.0285 for the ion [M-H-C_12_H_20_O_6_-H_2_O]^-^. These MS features led to the annotation of m35 and m36 as isograndidentatin A and grandidentatin A respectively, two compounds already identified in *P. alba* and *Populus ussuriensis* (Si et al., 2011; Tawfeek et al., 2019). Compound m39 presented the ion [M-H]^-^ at *m*/*z* 431.1334 and was putatively identified as populoside B, as previously described in *Populus davidiana* (Zhang et al., 2006). Compound m42 presented the following ions in ES^−^; [M-H]^-^ at *m*/*z* 543.1494, [M-H + formic acid]^-^ at *m*/*z* 589.1545, [M-H-C_7_H_6_O_3_]^-^ at *m*/*z* 405.1171 and [M-H-C_7_H_6_O_3_-H_2_O]^-^ at *m*/*z* 387.1093. Based on this fragmentation pattern and consistency with previous work on *Homalium ceylanicum*, this compound was putatively identified as homaloside D (Boeckler et al., 2013; Ekabo and Farnsworth, 1993). Compounds m43 and m44 displayed similar molecular ion masses, with [M-H]^-^ ions at *m*/*z* 563.1772 and 563.1769, and corresponding [M-H + formic acid]^-^ ions at *m*/*z* 609.1820 and 609.1807, respectively. Fragment ions at *m*/*z* 269.0812 and 269.0818 resulting from the sequential losses of a pentoside and hexoside [M-H-C_5_H_8_O_4_-C_6_H_10_O_5_]^-^ revealed an aglycone identified as pinostrobin, a salicinoid previously described in *P. nigra* (Ristivojević et al., 2015; Rubiolo et al., 2013). Accordingly, compounds m43 and m44 were both putatively identified as two isomers of pinostrobin pentoside hexoside. Compounds m46 and m47 presented respective [M-H]^-^ ions at *m*/*z* 431.1334 and 431.1371, as well as [M-H-C_6_H_10_O_5_]^-^ ions at 269.0817 and 269.0811 revealing two aglycones pinostrobin. These two compounds were putatively identified as two isomers of pinostrobin hexoside. Compound m15 presented ion [M-H]^-^ at *m*/*z* 577.1319, characteristic of proanthocyanidin dimer. Compounds m24 and m29 presented similar MS spectra with respective [M-H]^-^ ions at *m*/*z* 477.1050 and 477.1030. Both were putatively identified as isomers of isorhamnetin hexoside based on accurate mass and retention times from previous descriptions in *Populus* species (Danise et al., 2021; Pobłocka-Olech et al., 2021). Compound m25 produced the ion [M-H]^-^ at *m*/*z* 593.1486, corresponding to the previous identification of kaempferol rutinoside in *P. alba* (Danise et al., 2021).

Compound m45, present in the extract at low concentration, produced the ions [M-H]^-^ at *m*/*z* 389.1228 in negative mode. Based on MS data and retention time, compound m45 was putatively annotated as populin, a salicinoid previously reported in various *Populus* species (Pobłocka-Olech et al., 2021; Van Hoof et al., 1989).

MS/MS data for metabolites m9, m14, m17, m18, m23, m32, m34, m38, m40 and m41 were compared with MS/MS experimental data of several databases, including GNPS, but did not lead to their identification (level 4 of identification) (Supplementary Table S1).

### Metabolite concentration and productivity of *P. nigra* shoots grown in Magenta™ and RITA® systems

After establishing the metabolome of *P. nigra* shoots, phenolic productivity was compared between the two *in vitro* culture systems: Magenta™ and RITA®. Absolute concentrations of 17 metabolites were determined after 2 weeks of growth in Magenta™, corresponding to the subculture frequency (data not shown), and after 3 weeks in RITA®, which represents the optimal growth period determined from growth kinetic experiments (Figure 2). Productivity was expressed in mg/week, allowing comparison of the two *in vitro* systems based on biomass accumulation and metabolite concentration. The concentrations and productivity of the major phenolics, namely, 5-caffeoylquinic acid, salicin, PAC B1, PAC B3, catechin, 3-caffeoylquinic acid, caffeic acid, epicatechin, PAC B, coumaric acid, rutin, hyperoside, isoquercitrin, salicortin, quercitrin, nigracin and populoside were analyzed (Supplementary Table S2).

The concentrations of PAC B1, PAC B3 and populoside were significantly higher in Magenta™ system compared to RITA® with increases of 45%, 64% and 23% respectively. In contrast, 3-caffeoylquinic acid and rutin accumulated to a greater extent in RITA® with increases of 24% and 82% respectively. The remaining compounds, as well as the total phenolic content (sum of these 17 metabolites), were not significantly different between the two systems.

However, in terms of productivity, total phenolics were substantially higher in the RITA® system, showing a 160% increase relative to Magenta™. While the productivities of caffeic, coumaric acid and quercitrin were similar in both systems, most other metabolites were produced more efficiently in RITA®. For instance, productivity of 5-caffeoylquinic acid was increased by 218%, salicin by 167%, PAC B1 by 92%, PAC B3 by 71%, catechin by 185%, 3-caffeoylquinic acid by 241%, epicatechin by 150%, PAC B by 120%, rutin by 420%, hyperoside by 380%, isoquercitrin by 250%, salicortin by 133%, nigracin by 329% and populoside by 120%. This enhanced productivity was primarily attributed to the greater biomass accumulation achieved in the RITA® system compared to Magenta™.

### Metabolite composition of *P. nigra* shoots during growth in RITA®

**FIGURE 5:**
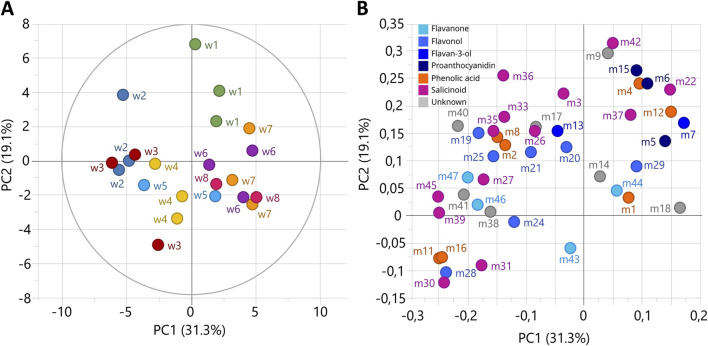
Unsupervised classification using principal component analysis (PCA) based on the relative concentration of metabolites during the 8 weeks of *Populus nigra* shoot growth in RITA® systems. **(A)** Score plot with colors according to growth weeks. **(B)** Loading plot of 45 metabolites, colored according to the polyphenol class. Numbers refer to the ID of compounds given in Figure 4.

The metabolic composition of *P. nigra* shoots grown in RITA® system was investigated over 8 weeks of culture by targeted metabolomics analyses. A principal component analysis on the relative concentration of 47 metabolites was performed as unsupervised analysis (Figure 5). The first two principal components explained 50.4% of the variation and highlighted metabolic changes occurring during the 8 weeks of culture in RITA® system. The first week of growth was projected on PC2-positive score, the second and third weeks on PC1-negative score, and the sixth, seventh and eighth weeks on PC1-positive score (Figure 5A). The fourth and fifth weeks mark a transitional state between the third and sixth weeks. On the loading plot, proanthocyanidins and catechin, as well as the salicinoids salicortin, populoside, homaloside D and the phenolic acids caffeic acid hexoside and ferulic acid hexoside were projected on PC2-positive score and thus mostly accumulated during the first week of growth (Figure 5B). Most of the remaining compounds were projected on PC1-negative score and correlated with the second and third weeks of growth. The fourth, fifth, sixth, seventh and eighth weeks of growth did not accumulate specific compounds.

### Elicitation in temporary immersion bioreactors for induced biomolecule production

Three-week-old RITA® cultures of black poplar were elicited with 100 µM of MeJa or SA for 1–4 days. Targeted metabolomic analyses were performed on the corresponding elicited extract and multivariate statistical analysis was performed to highlight metabolic changes during elicitations.

**FIGURE 6:**
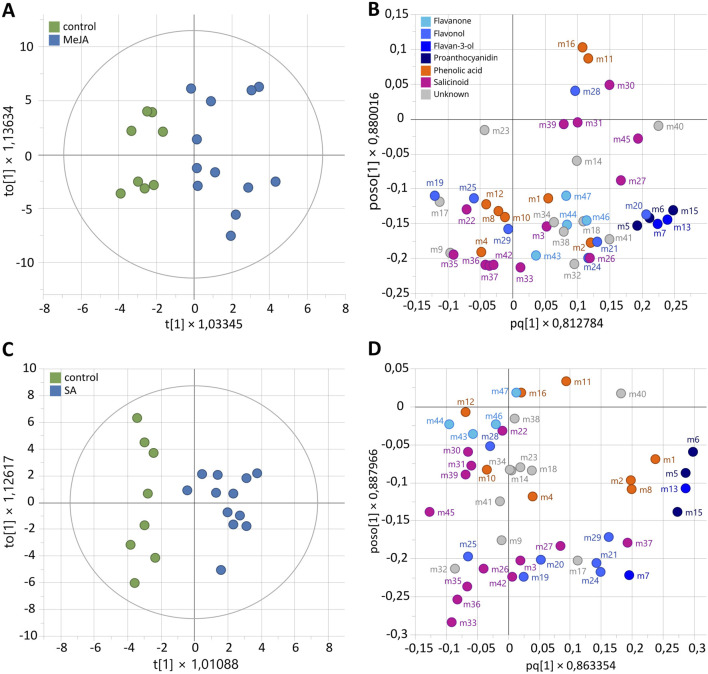
Supervised classification using orthogonal partial least squares discriminant analysis (OPLS-DA) based on the relative concentration of metabolites during elicitation of *Populus nigra* shoot growth in RITA® systems with elicitation status as discriminant variable, using 100 µM MeJA **(A,B)** or SA **(C,D)**. **(A,C)** Score plots. **(B,D)** Loading plots. Numbers refer to the ID of compounds given in Figure 4.

A first Orthogonal Partial Least Squares Discriminant Analysis (OPLS-DA) was performed with MeJA-elicitation as a discriminant variable. OPLS-DA model (diagnostic: R^2^ Xcum = 50.8%, R^2^Ycum = 76.1% and Q^2^cum = 76.1%) showed two well-separated groups according to the MeJA treatment (Figures 6A,B). Biomarkers of MeJA elicitation with Variable Importance (VIP) > 1 corresponded to flavan-3-ols (m7 catechin, m13 epicatechin), proanthocyanidins (m5 PAC B1, m6 PAC B3, m15 PAC B) and the flavonol hyperoside (m20). A second OPLS-DA model was performed with SA-elicitation as a discriminant variable. The OPLS-DA model (diagnostic: R^2^Xcum = 37.1%, R^2^Ycum = 87.4% and Q^2^cum = 61.8%) displayed two distinct groups according to SA treatment (Figures 6C,D). Biomarkers of SA elicitation with VIP >1 were the flavan-3-ol epicatechin (m13) and proanthocyanidins (m5 PAC B1, m6 PAC B3, m15 PAC B) (Figure 6D). With four metabolites induced in both cases and only two metabolites (catechin and hyperoside) specifically induced with MeJa, the two elicitors appear to have a similar effect.

**FIGURE 7:**
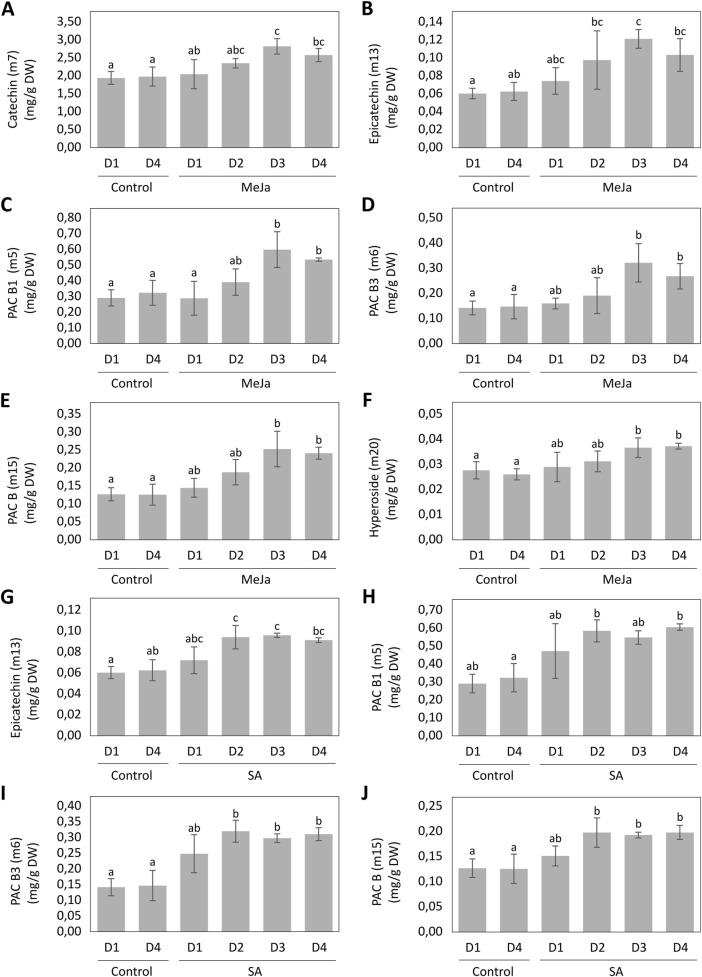
Induction kinetics of biomarkers of MeJA **(A–F)** and SA **(G–J)** elicitations with Variable Importance (VIP) > 1 of the OPLS-DAs models, during 4 days of elicitation. Different letters indicate significant differences (p < 0.05).

Univariate statistics were performed on selected biomarkers after MeJA and SA elicitation. Following elicitation with 100 µM of MeJa, catechin, epicatechin, PAC B1, PAC B3, PAC B and hyperoside reached their highest concentrations after 3 days (Figures 7A–F). Catechin was induced by 43%, epicatechin by 94%, PAC B1 by 85%, PAC B3 by 118%, PAC B by 101% and hyperoside by 41% in comparison with control cultures, reaching concentrations of 2.82 ± 0.22, 0.12 ± 0.01, 0.60 ± 0.11, 0.32 ± 0.08, 0.25 ± 0.05 and 0.04 ± 0.004 mg/g DW respectively. Epicatechin, PAC B1, PAC B3 and PAC B reached their highest concentrations after 2 days of elicitation with 100 µM SA (Figures 7G–J). Epicatechin was induced by 56%, PAC B1 by 101%, PAC B3 by 126% and PAC B by 56% in comparison with control cultures, reaching concentrations of 0.09 ± 0.01, 0.58 ± 0.06, 0.32 ± 0.03 and 0.20 ± 0.03 mg/g DW, respectively.

## Discussion

Black poplar extracts exhibit a broad range of biological activities with promising pharmaceutical and cosmetic applications. These biological activities are attributed to their rich composition in specialized metabolites, including salicinoid, flavan-3-ols, proanthocyanidins, phenolic acids and flavonols. Notably, antioxidant activities have been reported for several compounds such as caffeic and coumaric acids, isograndidentatin A, grandidentatin A, populoside, rutin and quercitrin (Dudonné et al., 2011; Li X et al., 2016; Pobłocka-Olech et al., 2021; Si et al., 2009). In addition, proanthocyanidins, catechin and quercitrin have demonstrated anti-inflammatory activities (Tang et al., 2019; Terra et al., 2007). Moreover, both catechin and rutin exhibit antimicrobial properties (Orhan et al., 2010; Veluri et al., 2004). Among the salicinoids, nigracin, one of the main compounds in *P. nigra* extracts, has been shown to promote fibroblasts growth and support tissue repair (Sferrazza et al., 2020). The purpose of this study was to investigate the potential of black poplar *in vitro* shoot cultures grown in temporary immersion bioreactors, with the aim of producing *P. nigra* bioactive extracts. After initiation and selection of *in vitro* lines, untargeted metabolomic analyses by UPLC-ToF-MS were carried out for confident identification of metabolites. The advantage of phenolic productivity in RITA® system was compared to Magenta™. Targeted metabolomics followed by PCA analysis allowed to study the metabolic changes during 8 weeks of culture in RITA® system. Finally, the effects of both SA and MeJA treatments on the metabolome of *P. nigra* were characterized by OPLS-DA. The *in vitro* shoot extracts contained 15 compounds described for the first time in *P. nigra*, along with 17 additional compounds not previously identified in the *Populus* genus. Optimal shoot growth was achieved in RITA® system after 3 weeks of culture. Treatments with MeJa and SA stimulated the accumulation of flavan-3-ols, proanthocyanidins and one flavonol, resulting in concentrations exceeding those reported in wild *P. nigra*. In contrast, phenolic acids, salicinoids and the remaining flavonols were not induced and exhibited lower concentrations compared to natural black poplar populations.

### Growth in RITA® system

In RITA® system, the highest growth index (5.5-fold) was observed for PN5 line after 3 weeks of culture system. As this study represents the first report on cultures of *P. nigra* cultures grown in RITA® system, the absence of equivalent studies limits direct comparisons of growth performance. However, other studies have reported biomass increases of 3.2-fold in plantain (*Musa* AAB), 5.5-fold in *Stevia rebaudiana* Bertoni, 3.75-fold in *Rhododendron tomentosum*, and 4.4-fold in *Salvia apiana* shoots after 3 weeks of growth in temporary immersion bioreactors (Aragón et al., 2005; Christela Melviana et al., 2021; Jesionek et al., 2017; Krol et al., 2023). Therefore, the growth index observed in *P. nigra* shoots falls in the same range as those previously reported for other species.

After 2 weeks of growth in RITA® system, browning of the culture medium became apparent. Browning in *in vitro* cultures was shown to negatively affect biomass growth and is regarded as a major challenge in plant and tissue culture (Abohatem et al., 2011; Li Y et al., 2016). This phenomenon is often observed in *in vitro* cultures of *Populus* species and other woody plants (Laffon et al., 2024; Thakur et al., 2012). The browning results from intrinsic factors such as species and genotype, or from external factors including the composition of the culture medium (Han et al., 2010; Rezaei-Moghadam et al., 2011; Xu et al., 2023). One possible explanation for this phenomenon is non enzymatic browning, which results from Maillard reactions and carbohydrates pyrolysis occurring at high temperatures (Chen et al., 2019). A second explanation is enzymatic browning, caused by the oxidation of phenolic compounds catalyzed by enzymes such as polyphenol oxidase (PPO) and peroxidase (POD) (Vaughn and Duke, 1984). In this process, polyphenols are oxidized in quinones, highly reactive and unstable intermediates, leading to the formation of complexes and polymers and then to brown pigments (Vámos-Vigyázó and Haard, 1981). Following subculture, which involves mechanical injury at the cutting site, phenolic compounds are released from the vacuole and become available for enzymatic oxidation (Mayer and Harel, 1979). Additionally, the activation of the phenylpropanoid pathway has been shown to promote POD biosynthesis, contributing to browning (Ackah et al., 2022; Yang et al., 2023). In this study, significant browning of the culture medium was observed between the second and fourth week of culture, occurring simultaneously with the highest production of phenolic compounds and, therefore, likely linked with activation of phenylpropanoid pathway.

### Metabolome of *P. nigra* shoots grown *in vitro*

*In vitro* explants of *P. nigra* grown in RITA® system exhibited notable differences in their phenolic profiles compared to previous reported data for this species. For instance, caffeic acid, coumaric acid, 5-caffeoylquinic and 3-caffeoylquinic acid were previously identified in *P. nigra* buds, but not in other organs (Dudonné et al., 2011; Kis et al., 2021; Stanciauskaite et al., 2021). Ferulic acid was described in both buds and leaves (Dudonné et al., 2011; Kurkin et al., 2020), but its glucoside form has not been documented in *P. nigra* or any other species within the *Populus* genus. Likewise, dihydroxybenzoic acid has been described in *P. nigra* buds*,* but its glucoside derivative was never identified in the genus (Kis et al., 2021). While coumaric acid glucoside and caffeic acid hexoside have been previously detected in *P. tremula* x *P. alba* hybrids (Morse et al., 2007; Saleme et al., 2017), this study is the first to report their presence in *P. nigra*. These findings suggest that shoots cultivated in RITA® system may have an enhanced capacity to accumulate glycosylated metabolites, a mechanism to protect the plant from toxic aglycones (Zhang W et al., 2022).

Flavan-3-ols catechin and epicatechin, as well as proanthocyanidin B1 were previously identified in *P. nigra* (Ullah et al., 2019a). However, proanthocyanidin B3 was only identified in other Populus species, such as *P. trichocarpa* (Li et al., 2025). Another flavan-3-ol dimer was detected but could not be fully annotated (m17).

The salicinoids salicin, salicortin and nigracin were already identified in leaves and bark of *P. nigra* (Palo, 1984; Pobłocka-Olech et al., 2021). Salicin was also detected in buds of *P. nigra* (Dudonné et al., 2011). Populoside, its isomer populoside A, and populoside B were identified in the bark of *P. davidiana* and leaves of *Populus deltoides* (Tschaplinski et al., 2019; Zhang et al., 2006). Other populoside isomers were detected in buds of several *Populus* species, but never in *P. nigra* (Okińczyc et al., 2024). Grandidentoside was identified in wood and bark of *P. tremula* and *Populus grandidentata* (Abreu et al., 2020; Erickson et al., 1970). Trichocarposide was identified in bark of *P. deltoides* (Pearl and Darling, 1971). Grandidentatin was detected in several *Populus* species, such as *P. davididana*, *P. grandidentata* x *P. alba*, *P. tremula*, *P.tremuloides*, *P.alba* and *P.ussuriensis* (Coleman et al., 2008; Palo, 1984; Si et al., 2011; Tawfeek et al., 2019; Zhang et al., 2006). However, isograndidentatin A was only identified in the bark of *P. ussuriensis* (Si et al., 2011). Homaloside D, the major compound in *P. nigra* extracts, was previously identified in *P. nigra* and seems to be specific to this species (Boeckler et al., 2013). Finally, populin was detected in *P. berolinensis*, *P. canadensis* ‘Marilandica’ and *P. trichocarpa* x *P. deltoides* ‘Beaupré’, but not yet in *P. nigra* (Pobłocka-Olech et al., 2021; Van Hoof et al., 1989).

Regarding flavonols, rutin, hyperoside, isoquercitrin, isorhamnetin glucoside and quercitrin, these compounds were already identified in *P. nigra* leaves (Pobłocka-Olech et al., 2021). Metabolites m24 and m29 were annotated as isorhamnetin glucosides, but the lack of standards and close retention time complicates definitive structural identification. Kaempferol rutinoside and isorhamnetin hexoside were both previously detected in *P. alba* (Danise et al., 2021).

This study represents the first comprehensive metabolic profiling of *in vitro P. nigra* shoots grown in RITA® system. Overall, 15 compounds were previously identified in *P. nigra* (m2, m3, m5, m7, m8, m11, m13, m16, m19, m20, m21, m22, m28, m30 and m42), 15 compounds were detected in other *Populus* species but not in *P. nigra* (m4, m6, m10, m24, m25, m26, m27, m29, m31, m33, m35, m36, m37, m39 and m45), seven compounds were never described in the *Populus* genus (m1, m12, m15, m43, m44, m46 and m47), and 10 compounds remained unknown (m9, m14, m17, m18, m23, m32, m34, m38, m40 and m41). Further phytochemical investigations, including compound purification and structural characterizations by NMR, will be necessary to fully annotate the metabolome of *in vitro P. nigra* shoot. Nevertheless, the present results already demonstrate the unique chemical profile of RITA® -cultivated *P. nigra* extracts, and suggest the potential for discovering novel biological activities.

### Comparison of metabolite production between *in vitro* cultures and field-grown plants

The accumulation of specialized metabolites in black poplar was compared between solid cultures grown in Magenta™ vessels and liquid cultures in RITA® bioreactors. While the total concentration of phenolics was similar for both systems, the levels of individual compounds, including PAC B1, PAC B3, populoside, 3-caffeoylquinic acid, rutin and hyperoside varied between the culture methods. In the literature, the impact of RITA® transfer on specialized metabolism varies depending on the plants studied. For example, anthocyanin concentration in *Phalaenopsis amabilis* decreased after transfer to RITA®, whereas total alkaloid contents from *D. nobile*, as well as total phenolic contents from *V. flexuosa* increased following the transfer (Mohammadpour Barough et al., 2024; Park et al., 2015; Zhang B et al., 2022). Essential oil content was similar between solid and RITA® cultures of *S. apiana*, whereas in *R. tomentosum*, essential oil levels were over 3 times higher in RITA® cultures compared to Magenta™ vessels (Jesionek et al., 2017; Krol et al., 2023).

In this study, metabolite concentrations were comparable between Magenta™ and RITA® culture systems. However, biomass production was higher in RITA®, resulting in higher overall productivity. Therefore, *P. nigra* RITA® cultures appear well suited for efficient bioproduction of specialized compounds, in comparison with classical solid cultures.

Metabolic data from *in vitro* cultures were compared to concentrations found in the literature in field- or greenhouse-grown black poplar. Metabolic profiling revealed qualitative differences between *in vitro* cultures and natural populations of black poplar, while quantification using pure standards showed notable quantitative variations. The concentration of catechin in *in vitro* shoot extracts was higher than in leaves extracts and in similar range of stem extracts obtained from greenhouse-grown *P. nigra* (Ullah et al., 2019a; 2019b). Similar trends were observed for PAC B1 concentrations. Likewise, epicatechin contents from greenhouse-grown leaf extracts were lower than in the *in vitro* shoots (Ullah et al., 2019a). Although, PAC B3 has not yet been quantified in field or greenhouse-grown black poplar, the levels detected in this study exceeded those reported in leaves of other species such as *P. trichocarpa* (Li et al., 2025).

Rutin and quercitrin were quantified in *P. nigra* leaves from natural populations, with concentrations higher than those found in *in vitro* shoot extracts (Boeckler et al., 2013). Isoquercitrin was quantified only in wild *P. nigra* buds, where its levels were lower than in *in vitro* shoot extracts (Benedec et al., 2014). Hyperoside was not yet quantified in field- or greenhouse-grown black poplar.

Caffeic acid and *p*-coumaric acid were quantified in buds and bark from natural *P. nigra* populations, with concentrations much higher than in *in vitro* shoot extracts (Kukina et al., 2017; Stanciauskaite et al., 2021). Conversely, 3-caffeoylquinic acid levels in wild buds were lower than in *in vitro* shoot extracts (Stanciauskaite et al., 2021). To date, 5-caffeoylquinic has not been quantified in *P. nigra*.

Salicin concentration in *in vitro* shoots extracts was comparable to that found in leaves obtained from wild *P. nigra* populations (Pobłocka-Olech et al., 2021). Salicortin was quantified in greenhouse-grown *P.nigra* leaves and reached higher contents compared to *in vitro* shoots extracts (Lackus et al., 2020). Nigracin was quantified in leaves from field-grown *P. nigra* and resulted in higher contents than in *in vitro* shoot extracts (Walther et al., 2025). Populoside has not yet been identified or quantified in *P. nigra*. However, the extracts from this study revealed higher levels of populoside than those reported in leaves of other populus species such as *P. deltoides* (Tschaplinski et al., 2019).

Overall, extracts from RITA® -grown cultures exhibited higher contents of catechin, epicatechin, PAC B1, 3-caffeoylquinic acid and isoquercitrin compared to those from natural populations of black poplar. Conversely, contents of coumaric and caffeic acids, rutin, quercitrin, salicortin and nigracin were lower than in wild poplar extracts. While absolute concentration should allow us direct comparison, it should also be kept in mind that this data was collected independently and that variations in quantity might also result from technical differences such as extraction protocols. Therefore, in the future, it could be interesting to do a direct comparison between natural populations and RITA® -grown cultures of *P. nigra*. Nevertheless, the current data indicate quantitative and qualitative differences between the metabolite production of *in vitro* and wild poplar. Ultimately, these differences may hold novel biological activities in *P. nigra* extracts produced in RITA® bioreactors, which remain to be explored.

### Flavan-3-ol and proanthocyanidin induction after MeJa or SA treatments

The phytohormones MeJa and SA both act as key signaling molecules that trigger plant defense responses under environmental stress. When applied as elicitors, they can increase the production of specialized metabolites, and may even induce the biosynthesis of new compounds (Jeyasri et al., 2023). In poplar, the treatment of leaves with methyl salicylate, a SA derivative, led to the increase of defense-related enzyme activities and stimulated the production of several volatile compounds (Tang et al., 2015).

In this study, flavan-3-ols and proanthocyanidins were induced in response to elicitation with MeJa and SA. These two classes of flavonoids derive from the phenylpropanoid pathway and their biosynthesis is well characterized (Yu et al., 2023). In plants, their biological function is mainly the protection against biotic and abiotic stresses (Dixon and Sarnala, 2020). Consequently, extracts containing these compounds exhibit a wide array of biological activities, including antioxidant, anti-inflammatory or anti-microbial properties (Rippin et al., 2022). For instance, under high light stress and nitrogen deficiency, the induction of proanthocyanidins has been associated with enhanced antioxidant activity. Furthermore, transgenic poplar lines with elevated concentrations of condensed tannins showed increased resistance to oxidative stress (Gourlay and Constabel, 2019).

The increase of flavan-3-ols and proanthocyanidins in response to diverse stresses was previously observed in several *Populus* species. A study highlighted that condensed tannins were strongly accumulated in *P. tremuloides* after artificial defoliation (Osier and Lindroth, 2001). Another study showed that genes encoding enzymes of proanthocyanidin biosynthesis were upregulated after infection of hybrid poplar (*Populus trichocarpa x P. deltoides*) with the fungus *Melampsora medusae* (Miranda et al., 2007). Similarly, MYB134, a transcription factor co-induced with proanthocyanidin biosynthetic genes, as well as many phenylpropanoid and flavonoid genes, were overexpressed in poplar in response to wounding, fungal infection and UV-B exposure (Mellway et al., 2009). In a later study, the accumulation of flavan-3-ols and proanthocyanidins was reported in *P. nigra* after fungal infection (Ullah et al., 2017). To complete these results, levels of endogenous SA, jasmonic acid and abscisic acid were measured in black poplar leaves and rose after fungal infection (Ullah et al., 2022). This suggests that, unlike in many other plant species where MeJA and SA act antagonistically, they may have positive interactions in poplar. The treatment of leaves with an analog of SA led to decreased fungal growth and induction of catechin, epicatechin and PAC B1, as well as activation of many flavonoid pathway genes (Ullah et al., 2019a). Similar results were obtained in black poplar stems in response to fungal infection (Ullah et al., 2019b).

In this study, flavan-3-ols and proanthocyanidins were induced together after 2 and 3 days of elicitation with SA or MeJa. However, further research is needed to identify the specific signaling pathways by which MeJa and SA regulate the specific induction of flavan-3-ols and proanthocyanidins.

### Induction of phenolic acids, flavonols and salicinoids

In this study, phenolic acids and salicinoids were not induced in response to elicitation with MeJa or SA. Regarding the flavonols, only hyperoside was increased in elicited cultures. Similarly, in several studies, flavonols and phenolic acids from black poplar leaves were not impacted by herbivory attacks or SA treatments (Boeckler et al., 2013; Ullah et al., 2019a). However, several flavonoids, including quercetin and kaempferol glycosides were accumulated after UV-B radiation of greenhouse and field-grown *P. trichocarpa* (Warren et al., 2023).

Regarding *in vitro* cultures, the treatment of hybrid poplar (*P. trichocarpa x P. deltoides*) cell suspensions with polygalacturonic acid lyase or with fungal elicitors led to the activation of phenylpropanoid pathway with the rise of PAL and 4CL enzymes activities. However, the monitoring of induced compounds and their identification was not carried out (Moniz de Sa et al., 1992).

Most of the existing research on poplar has focused on salicinoid induction, a group of compounds specific to the Salicaceae family. In *P. nigra*, increases in salicin were observed in leaves damaged by several herbivores, whereas salicortin and homaloside D were not induced. An accumulation of JA was also reported in elicited leaves, while SA levels remained unchanged (Fabisch et al., 2019). The salicinoids tremulacin and salicortin were reported to increase in response to drought stress of *P. nigra* trees (Hale et al., 2005).

Nevertheless, several studies show no impact of elicitation on salicinoid biosynthesis. For example, no increase in salicinoids content was observed after artificial defoliation or herbivory attacks (Boeckler et al., 2013; Osier and Lindroth, 2001). Similarly, treatment of poplar leaves with SA analog did not induce salicinoid accumulation (Ullah et al., 2019a).

In poplar, and in other species from the Salicaceae family, salicinoids play a role as defense against herbivory attacks (Boeckler et al., 2011). Their biosynthesis pathway remains incomplete. Likewise, salicylic acid biosynthesis pathway in poplar is still unclear, despite being fully elucidated in other species like Arabidopsis (Ullah et al., 2023). In 2010, new evidence showed that cinnamic acid and benzoic acid were precursors of salicortin and salicin, but that salicin was not a precursor of salicortin, as mentioned previously (Babst et al., 2010). In a later study, the treatment of *in vitro P. davidiana* with MeJa led to the accumulation of benzoic acid, salicylic acid, and all the benzoate intermediates, but not salicin, also highlighting that an alternative pathway could be underlying (Park et al., 2017). Recently, the enzyme UGT71L1 was identified as essential for the salicinoid biosynthesis pathway (Fellenberg et al., 2020). UGT71L1 knockout plants accumulated less salicinoids, more JA and SA, and showed growth anomalies (Gordon et al., 2022). These results highlight the apparent lack of antagonism of the two phytohormones in poplar and the incomplete characterization of the salicinoid biosynthesis pathway. Poplars genetically modified to overexpress the MYB134 tannin regulatory gene produce a high content of proanthocyanidins and also accumulate less salicinoids (Boeckler et al., 2014). Similar results were obtained on several poplar hybrids overexpressing the MYB115 gene, underlining the possible competition for precursors in the biosynthetic pathways (James et al., 2017).

The contradictory results regarding induction of salicinoids, the lack of information on their biosynthesis pathway and on the function of SA in poplar, as well as the relationship of SA and JA, are the main obstacles for a complete understanding of the results in this study (Morse et al., 2007; Ullah et al., 2022). The fact that salicinoids remained constant in elicited cultures might be due to the concurrent induction of proanthocyanidins. However, the relationship between these different classes of compounds is still unclear, which currently hinders the development of effective elicitation strategies for salicinoid production.

The chemical composition of black poplar extracts obtained from *in vitro* cultures exhibited many differences compared to wild poplar extracts, including the detection of compounds not previously reported for this species, as well as potentially novel metabolites. Also, further structural identification using purification and NMR techniques is needed to complete the metabolome annotation. RITA® extracts were particularly rich in glycosylated metabolites, suggesting the accumulation of otherwise toxic specialized metabolites. Elicitation with MeJA and SA resulted in the induction of flavan-3-ols and proanthocyanidins, metabolites typically associated with biotic and abiotic stresses. In contrast, the contents of salicinoids, phenolic acids and most of the flavonols remained unchanged following elicitation. Salicinoids are specific to the *Populus* genus, and their biosynthetic pathway remains poorly understood which limits the success of elicitation strategies. The high levels of flavan-3-ols and proanthocyanidins, coupled with the low accumulation of salicinoids, and the overall metabolic variations between *in vitro* and wild poplar extracts, suggest a promising potential for the discovery of novel biological activities.

In conclusion, the integration of RITA® temporary immersion systems for optimized *in vitro* growth with targeted elicitation strategies to enhance metabolite biosynthesis led to a significant increase in the production of black poplar secondary metabolites, as demonstrated by comprehensive metabolomic profiling. These results provide strong evidence that plant biotechnological approaches offer a viable and sustainable alternative for the large-scale production of specialized metabolites. Furthermore, this study emphasizes the potential of temporary immersion systems as an efficient platform for organ and tissue culture at an industrial scale.

## Data Availability

The metabolomics data have been deposited to MetaboLights (Yurekten et al., 2024) repository with the study identifier MTBLS13131.
