# Supplementary material for: Enhanced bioproduction of phenolic compounds from Populus nigra L. using temporary immersion bioreactors and metabolomics
Source: Front Mol Biosci. 2025 Nov 24;12:1704160. doi: 10.3389/fmolb.2025.1704160 (PMC12682903; doi:10.3389/fmolb.2025.1704160)
Supplement: Supplementary file 1 [file DataSheet1.pdf]

Supplementary Material

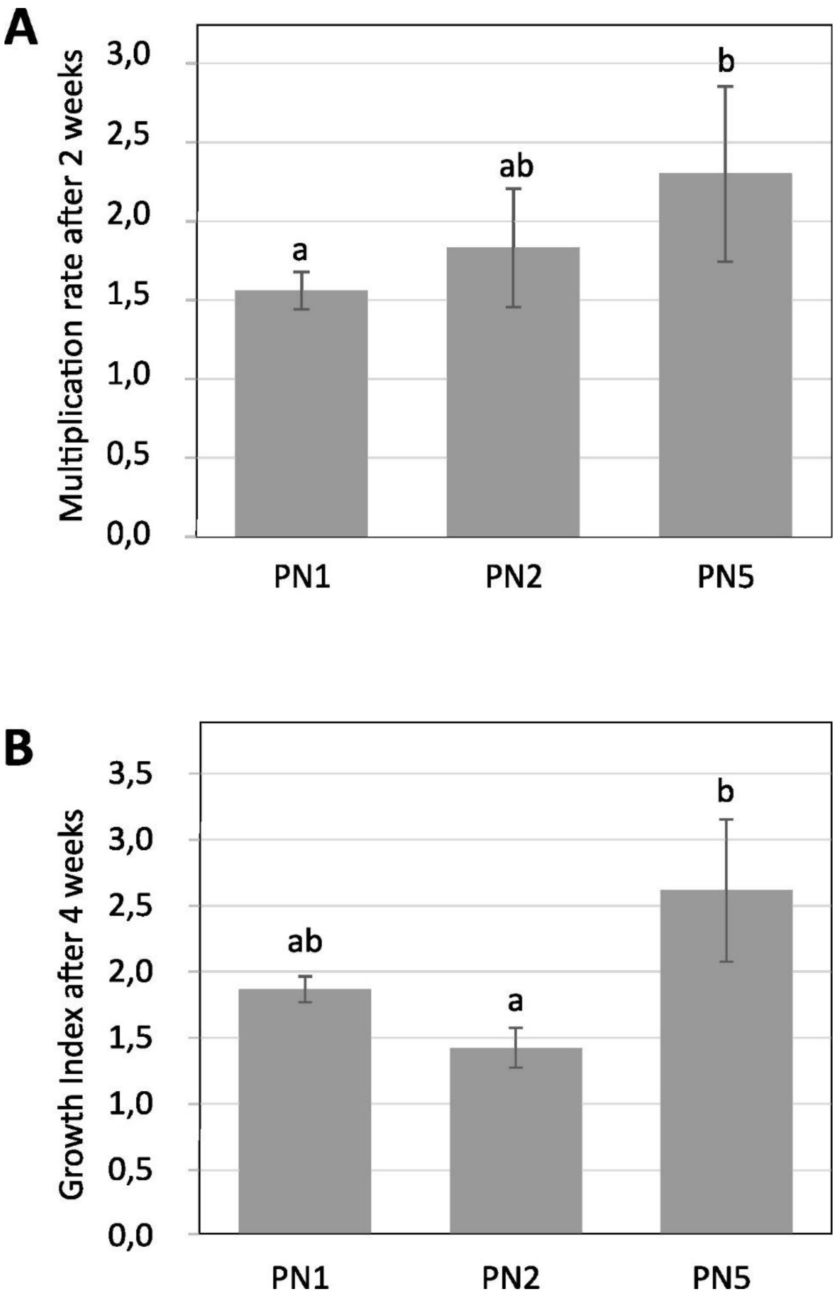

Supplementary Figure 1. Growth and multiplication of *P. nigra* lines PN1, PN2 and PN5. (A) Multiplication rate of the three lines grown in Magenta ™ systems after 2 weeks. (B) Growth index of the three lines grown in RITA® systems after 4 weeks. Different letters indicate significant differences ($p < 0.05$).

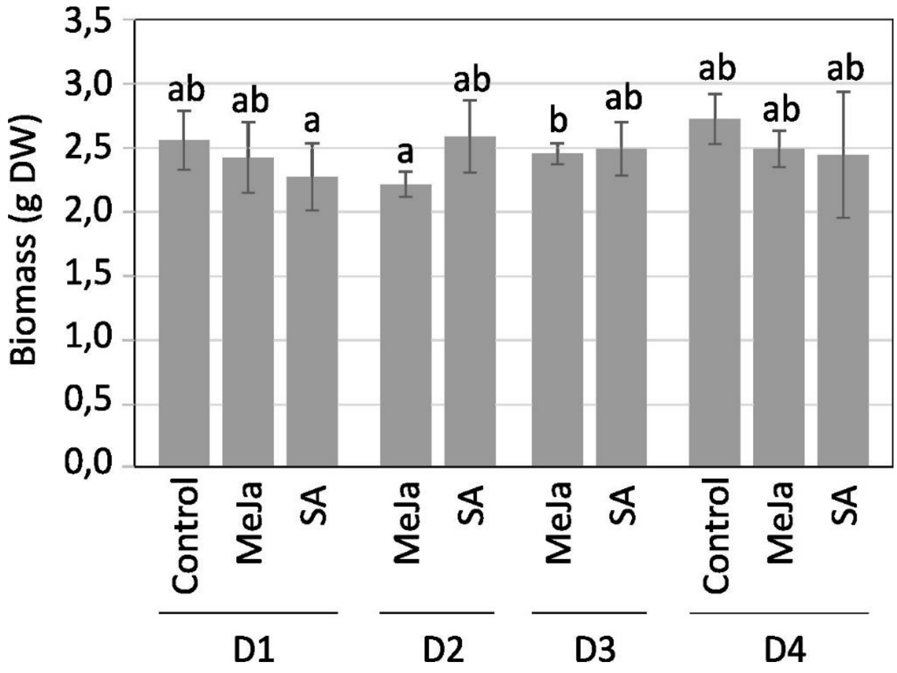

Supplementary Figure 2. Effect of 100 µM MeJa and SA treatments on *P. nigra* shoot biomass grown in Magenta ™ systems after 1, 2, 3 and 4 days. Different letters indicate significant differences ($p < 0.05$).

Supplementary Table 1. UHPLC-ESI-HRMS data of unknown compounds from *P. nigra* shoots grown in Magenta ™ systems. Numbers in the column "ID" refer to peak numbers in Figure 4.

ID	RT (min)	λ_{max} (nm)	Compound assignment	m/z measured	MS/MS fragments ES$^-$	Molecular formula	m/z calculated	Error (ppm)
**m9**	5.87	281	Unknown	301.0922		C13H18O8	301.0923	-0.47
**m14**	6.89	216	Unknown	401.1427		C18H26O10	401.1448	-5.17
**m17**	7.96	218	Unknown	369.1196		C17H22O9	369.1186	2,82
**m18**	8.53	218	Unknown	415.1235		C18H24O11	415.1240	-1.35
**m23**	9.51	218	Unknown	397.1466		C19H26O9	397.1499	-8.24
**m32**	11.19	219/317	Unknown	433.1113		C21H22O10	433.1135	-5.01
**m34**	11.5	218/321	Unknown	517.1330		C25H26O12	517.1346	-3.14
**m38**	12.1	219/324	Unknown	409.1498		C20H26O9	409.1499	-0.20
**m40**	12.57	220/315	Unknown	417.1165		C21H22O9	417.1186	-4.98
**m41**	13.15	220/281	Unknown	447.2245	315.1807 $[M-H+C_5H_8O_4]^-$	C21H36O10	447.2230	3.30

Supplementary Table 2. Concentrations (mg/g DW) and productivity (mg/week) of metabolites produced from *P. nigra* shoot cultures grown in Magenta™ and RITA® systems in comparison with field and greenhouse cultures. Different letters within a row indicate significant differences ($p < 0.05$).

Metabolite	Concentration (mg/g DW)			Productivity (mg/week)	
	Magenta ™	RITA®	Field/greenhouse*	Magenta ™	RITA®
5-Caffeoylquinic acid	0.34 ± 0.02 *a*	0.40 ± 0.07 *a*	NA	0.11 ± 0.01 *a*	0.35 ± 0.07 *b*
Salicin	2.97 ± 0.55 *a*	2.89 ± 0.65 *a*	3.01 ± 1.66[1]	0.94 ± 0.21 *a*	2.51 ± 0.68 *b*
PAC B1	0.42 ± 0.03 *b*	0.29 ± 0.05 *a*	0.091 ± 0.011[2]	0.13 ± 0.02 *a*	0.25 ± 0.05 *b*
PAC B3	0.23 ± 0.03 *b*	0.14 ± 0.03 *a*	NA	0.07 ± 0.01 *a*	0.12 ± 0.03 *b*
Catechin	1.87 ± 0.30 *a*	1.94 ± 0.18 *a*	0.29 ± 0.02[2]	0.59 ± 0.12 *a*	1.68 ± 0.22 *b*
3-Caffeoylquinic acid	0.54 ± 0.03 *a*	0.67 ± 0.06 *b*	0.26 ± 0.01[3]	0.17 ± 0.01 *a*	0.58 ± 0.08 *b*
Caffeic acid	0.06 ± 0.02 *a*	0.04 ± 0.03 *a*	1.68 ± 0.01[3]	0.02 ± 0.01 *a*	0.03 ± 0.03 *a*
Epicatechin	0.08 ± 0.02 *a*	0.06 ± 0.01 *a*	0.019 ± 0.005[2]	0.02 ± 0.01 *a*	0.05 ± 0.01 *b*
PAC B	0.17 ± 0.06 *a*	0.13 ± 0.02 *a*	NA	0.05 ± 0.02 *a*	0.11 ± 0.02 *b*
Coumaric acid	0.05 ± 0.01 *a*	0.05 ± 0.04 *a*	0.71 ± 0.01[3]	0.01 ± 0.01 *a*	0.05 ± 0.04 *a*
Rutin	0.33 ± 0.04 *a*	0.60 ± 0.10 *b*	3.4 ± 0.3[4]	0.10 ± 0.01 *a*	0.52 ± 0.11 *b*
Hyperoside	0.016 ± 0.005 *a*	0.028 ± 0.003 *b*	NA	0.005 ± 0.001 *a*	0.024 ± 0.003 *b*
Isoquercitrin	0.07 ± 0.02 *a*	0.08 ± 0.01 *a*	0.005 ± 0.0005[5]	0.02 ± 0.01 *a*	0.07 ± 0.01 *b*
Salicortin	7.22 ± 2.55 *a*	6.15 ± 1.95 *a*	50.19 ± 10.40[6]	2.24 ± 0.61 *a*	5.22 ± 1.48 *b*
Quercitrin	0.12 ± 0.07 *a*	0.05 ± 0.03 *a*	0.6 ± 0.1[4]	0.04 ± 0.02 *a*	0.04 ± 0.03 *a*
Nigracin	0.42 ± 0.20 *a*	0.68 ± 0.35 *a*	1.34 ± 0.05[7]	0.14 ± 0.07 *a*	0.60 ± 0.36 *b*
Populoside	0.16 ± 0.01 *b*	0.13 ± 0.02 *a*	NA	0.05 ± 0.01 *a*	0.11 ± 0.02 *b*
Total phenolics	15.05 ± 1.66 *a*	14.32 ± 1.60 *a*	-	4.73 ± 0.14 *a*	12.32 ± 1.34 *b*

*Data from the literature : [1] Pobłocka-Olech et al., 2021 ; [2] Ullah et al., 2019a ; [3] Stanciauskaite et al., 2021 ; [4] Boeckler et al., 2013 ; [5] Benedec et al., 2014 ; [6] Lackus et al., 2020 ; [7] Walther et al., 2025 ; NA = Not available.
